# How is ethnicity reported, described, and analysed in health research in the UK? A bibliographical review and focus group discussions with young refugees

**DOI:** 10.1186/s12889-023-16947-3

**Published:** 2023-10-17

**Authors:** Joseph Lam, Robert Aldridge, Ruth Blackburn, Katie Harron

**Affiliations:** 1 UCL Great Ormond Street Institute of Child Health, 30 Guilford St, London, WC1N 1EH UK; 2 Institute for Health Metrics and Evaluation, University of Washington, Seattle, WA 98195 USA; 3 UCL Institute of Health Informatics, 222 Euston Rd, London, NW1 2DA UK

**Keywords:** Racism, Ethnicity, Migrants, Administrative data, Data linkage

## Abstract

**Background:**

The ethnicity data gap pertains to 3 major challenges to address ethnic health inequality: 1) Under-representation of ethnic minorities in research; 2) Poor data quality on ethnicity; 3) Ethnicity data not being meaningfully analysed. These challenges are especially relevant for research involving under-served migrant populations in the UK. We aimed to review how ethnicity is captured, reported, analysed and theorised within policy-relevant research on ethnic health inequities.

**Methods:**

We reviewed a selection of the 1% most highly cited population health papers that reported UK data on ethnicity, and extracted how ethnicity was recorded and analysed in relation to health outcomes. We focused on how ethnicity was obtained (i.e. self reported or not), how ethnic groups were categorised, whether justification was provided for any categorisation, and how ethnicity was theorised to be related to health.

We held three 1-h-long guided focus groups with 10 young people from Nigeria, Turkistan, Syria, Yemen and Iran. This engagement helped us shape and interpret our findings, and reflect on.

1) How should ethnicity be asked inclusively, and better recorded?

2) Does self-defined ethnicity change over time or context? If so, why?

**Results:**

Of the 44 included papers, most (19; 43%) used self-reported ethnicity, categorised in a variety of ways. Of the 27 papers that aggregated ethnicity, 13 (48%) provided justification. Only 8 of 33 papers explicitly theorised how ethnicity related to health.

The focus groups agreed that 1) Ethnicity should not be prescribed by others; individuals could be asked to describe their ethnicity in free-text which researchers could synthesise to extract relevant dimensions of ethnicity for their research; 2) Ethnicity changes over time and context according to personal experience, social pressure, and nationality change; 3) Migrants and non-migrants’ lived experience of ethnicity is not fully inter-changeable, even if they share the same ethnic category.

**Conclusions:**

Ethnicity is a multi-dimensional construct, but this is not currently reflected in UK health research studies, where ethnicity is often aggregated and analysed without justification. Researchers should communicate clearly how ethnicity is operationalised for their study, with appropriate justification for clustering and analysis that is meaningfully theorised. We can only start to tackle ethnic health inequity by treating ethnicity as rigorously as any other variables in our research.

**Supplementary Information:**

The online version contains supplementary material available at 10.1186/s12889-023-16947-3.

## Background

Ethnicity refers to a multi-dimensional social construct encompassing language, country of origin, cultural heritage, nationality and more [1]. Since the 1991 Great Britain Census, ethnicity in the UK has been conceptualised as a subjective, self-defined construct, and commonly operationalised in research and administrative data under hierarchical ethnic categories with “Asian”, “Black”, “White”, “Mixed” and “Other” as the 5 high-level ethnic groups [2–4]. Reducing racial and ethnic health inequities has long been a priority for health policy in the UK [5]. However, ethnic health inequity remains prominent across medical disciplines, from stillbirth rates, access to mental health treatments, to cancer incidence and excess mortality from Coronavirus Disease 2019 (Covid-19)[6–9]. The National Healthcare Service Race and Health Observatory report [10] cites “lack of good-quality data and analysis” as the main barrier to addressing ethnic health inequities.

The ethnicity data gap reflects three interlinked issues [11]; i) the under-representation of people from minoritized ethnic groups in health research, ii) inconsistent and poor quality recording of ethnicity in health and administrative data and iii) the rigid, hierarchical ethnic categories used by researchers when analysing ethnicity that might not represent individuals’ and groups’ ethnic identity. In addition, how researchers analyse ethnicity data, and report (or omit) results of analyses of ethnicity, adds a further layer of complexity; 40% of randomised controlled trials funded by the National Institute for Health and Care Research did not report on ethnicity at all [12].

### Under-representation of minoritized ethnic groups

The under-representation of minoritized ethnic groups is evidenced by human genetics research that uses a majority sample of European heritage, such as the UK Biobank (94% White), and is therefore not generalisable to UK populations (82% White) [13–16]. An alternative approach is to use linked population-based data sources that capture whole populations. There are an increasing number of health administrative data linkage studies aiming to improve diversity in the research samples, for example in the study of population vaccine uptake and variant phenotypes during the pandemic [17, 18]. However, linkage studies merely replicate data quality issues in the source datasets [19], which disproportionately affect minoritized groups [20, 21].

### Quality of recording of ethnicity data

Issues of data quality may stem from the absence of guidelines on the collection of data on ethnicity and the breadth of collection approaches used (e.g. ethnicity being inferred from appearance, rated by healthcare workers based on proxy markers of ethnicity such as nationality, self-reported in fixed categories, or simply not collected). Selection biases may be amplified by approaches such as linkage, as minoritized ethnic populations are more likely to be missed and excluded in the process [22], and there are a variety of approaches to deal with inconsistent recording of ethnicity across linked data sources, or within a data source over time, despite this having important implications for results. For example, a recent study found the association between ethnicity, education attainment and neurodevelopmental disorder depends on which data source is used to primarily code ethnicity [23].

### Ethnicity as a construct in research

Ethnicity is often analysed as a static construct, contradictory to its subjective, multidimensional and dynamic nature [24]. A large body of literature on longitudinal studies, health records and administrative data, demonstrates that people’s ethnic group identity changes over time and context, with more variation for non-white than white populations [21, 25, 26]. Within most population health studies, variation in recording of ethnicity is inadvertently treated as error. This has motivated substantive methodological work in harmonising these inconsistencies, for example using the most recent or most common (modal) ethnicities, or other weighted algorithms [27, 28]. These approaches remain insensitive to the dynamic nature of ethnic identity across time and contexts within local populations [11].

As the UK welcomes an increasingly diversifying population, there is a strong case for existing ethnic categories to be updated to reflect community identity. Whist recent census waves increased number of ethnic categories in the survey, from 9 categories in the first survey in 1991 to 19 categories in the 2021 census, existing administrative and health record systems have yet to catch up with such changes. Comparisons of UK Census data over the last 30 years identified a sizable increase in number of people identified as “mixed” and “other” groups [29]. The evidence base for these groups is often limited, as researchers struggle to understand what “mixed” and “other” mean [30]. The theoretical framework of a static ethnic identity faces conceptual and analytical challenges.

### Meaningful analysis of ethnicity data

There is an urgent need for researchers to clarify how ethnicity is analysed in health research. In public health policy making, ethnic categories are rightfully used to foster comparisons to elucidate inequalities and prioritise resource distribution. However, the meaning of these categories is seldom made transparent, or communicated well to the public [11, 31]. What do we mean when we demonstrate that black women are 3.7 times more likely to die in pregnancy and childbirth than white women [32]? What do we mean when we model ethnicity as the cause of elevated risk for health outcomes? Theoretical frameworks for ethnicity in health have long been studied, from Nazroo’s [33] seminal work on how racism is fundamental to understanding ethnic health inequalities; Phelan and Link [34] describing how racism causes health inequalities independent of and in conjunction with social economic deprivations; Jongsma and colleagues [35] summarising the theoretical underpinning of ethnicity in relation to risks of psychotic disorders; to Bécares and colleagues [36] emphasis on racism acting at the structural, institutional, community and individual-levels leading to ethnic inequities in covid-19 vaccine hesitancy. It is less clear whether health researchers have been actively seeking to use and refine these theoretical models of ethnicity to help translate research into actionable health policies.

### Reporting of ethnicity data

In 2021, the JAMA Network published updated guidance on reporting race and ethnicity in their journal, mandating authors to report how and from what data source ethnicity was classified, and what categories were used [37]. They encourage authors to explicitly theorise how ethnicity relates to health outcomes. Whilst some research has explored heterogeneity in reporting of race/ethnicity, such as the different ethnic categories used in the study of long-term conditions [38], most reviews on ethnicity and health still focus on describing health outcomes stratified by ethnicity and rarely report on how ethnicity is analysed. There is also limited involvement from the public in shaping how ethnicity is captured, and how research findings are interpreted to shape health policy [11].

The purpose of this bibliographical review is to examine the ways ethnicity is theorised, captured, reported, categorised and analysed in top-cited health research in the UK context. We also aimed to explore what factors researchers should consider when studying ethnicity in relation to health. We organised 3 focus group discussions with a group of young migrants and refugees from Coram Young Citizens to help us shape our study and interpret our findings.

## Methods

1. Bibliographical review

This review is not pre-registered as PROSPERO does not accept methodological reviews that assess only the quality of reporting. This review followed a systematic approach outlined in the PRISMA statement where applicable [39].

### Eligibility Criteria

#### Inclusion

This review included empirical research from cross-sectional or longitudinal cohort studies or randomised controlled trials conducted in the UK, which was peer-reviewed, published, written in English, and reported ethnicity and any health-related outcomes.

#### Exclusion

We excluded reviews, studies including non-UK samples, studies that did not include ethnicity information, or those that did not include any health-related outcomes.

The search strategy is outlined in full in Additional file 1. We searched MEDLINE and Web of Science database in July 2022, covering published literature since 1946. Given the vast literature on ethnicity and health, we focused on the top 1% of papers according to number of citations for 3 separate timeframes: 1946–2000, 2001–2019, 2020–2022. We expected these papers to have a wider impact on how ethnicity is described and reported in other population health papers. The timeframes were split to mitigate temporal differences in the accumulation of citations, and changing publication rates, particularly for covid-related papers from 2020.

The number of citations for each paper was extracted from Google Scholar (see Additional file 2 for dates of search and records). Full texts were assessed for eligibility from most cited until the target number of papers had been reached.

### Data synthesis plan

Our data extraction strategy is outlined in Table 1. We planned to describe whether there was a consistent approach in capturing ethnicity across studies and data type, in particular how missing, mixed, other ethnic groups were treated, and how ethnic groups were aggregated. We then described the theoretical basis in which ethnicity was captured and analysed.

- 2) Public engagement / Focus Group Discussions

**Table 1:** Data extraction strategy

Data Extraction Strategy	Options	Notes
Data Source Type	Electronic health records, linked health data study, cross-sectional survey, longitudinal cohort, randomised controlled trials	
How was ethnicity asked and reported? If self-reported, how many categories?	Self-reported, prescribed, based on medical records, other	We separated ethnicity reported in medical records from self-reported ethnicity given the known inconsistencies of how ethnicity is asked in medical settings [21]
How were missing, mixed, and other ethnic groups described?	Whether other, missing and mixed are reported or included in analysis	
Was ethnicity aggregated in description or analysis?	Yes, No	
If so, were justifications provided?	Yes (describe), No	Justification is defined very broadly A simple one sentence explanation on why ethnicity is aggregated is sufficient to be classed as having provided a justification. We do not evaluate the quality of the justifications
How was ethnicity used in analysis?	Descriptor, predictor, other	
Was ethnicity conceptualised or theorised to link with health outcomes?	Yes, No	Only applicable to papers using ethnicity as a predictor Conceptualisation is defined very broadly. A simple one sentence description on how ethnicity is theorised to associate with health outcome is sufficient to be classed as having provided a theory

In collaboration with Coram Young Citizens, we organised 3 1-h-long guided focus groups. All materials were shared with 2 young people before finalised to ensure the questions and language used are appropriate. Materials we used for the focus groups are described in Additional file 3. Through a series of interactive games and discussions, we reflected on:

1. What is ethnicity? How should ethnicity be asked inclusively, and better recorded?
2. Does ethnicity change over time or context? If so, why?

Initial findings from this review were shared with the group for feedback and interpretation. As public engagement work, we did not use any specific research methods within our focus group discussions. However, we consolidated feedback from these sessions into a set of recommendations for researchers to study ethnicity and health.

## Results

We identified 3,200 records from MEDLINE and 1,546 records from Web of Science, where 412 were flagged as duplicates by Zotero. Of the 4,334 deduplicated papers, 290 were published between 1946–2000, 2,874 were published between 2001–2019, and 1,170 were published between 2020-July 2022. We rounded up the number of papers to be included in the review from each timeframe to include 3, 29, and 12 papers respectively, adding up to 44 papers to be synthesised (described in Additional file 4).

**Fig. 1:**
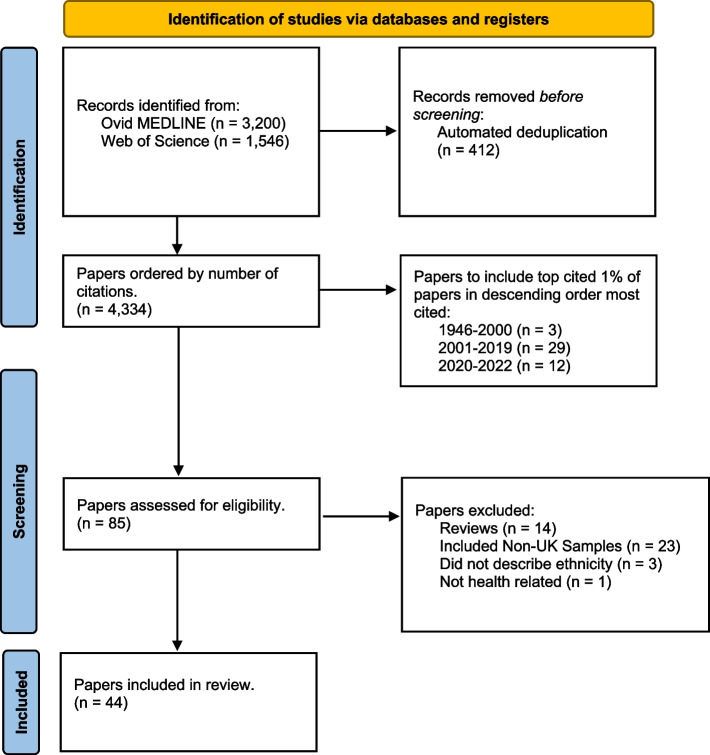
Flow diagram for the identification, screening, eligibility, and inclusion of studies [39]

Of 85 papers assessed for eligibility, 14 were excluded as they were non-empirical studies, 23 included non-UK samples, 3 did not describe ethnicity, and 1 did not include health related outcomes (Fig. 1). The 44 remaining papers involved samples from 40 unique studies based in the UK. Of these, 7 (15.9%) used electronic health records, 6 (13.6%) used linked health records with administrative data, 7 (15.9%) used cross-sectional survey, 23 (52.3%) were cohort studies, and 1 (2.27%) was a randomised controlled trial (Table 2).

**Table 2:** Ethnicity data source by timeframe of publication

Data Source	1946–2000	2001–2019	2020–2022
Total Number of Papers	3	29	12
Electronic health records	0	4 (13.8%)	3 (25%)
Linked health administrative data	0	2 (6.9%)	4 (33.3%)
Cross-sectional survey	0	6 (20.7%)	1 (8.3%)
Longitudinal Cohort (including birth cohort)	2 (66.7%)	17 (58.6%)	4 (33.3%)
Randomised Controlled Trials	1 (33.3%)	0	0

### Sources of ethnicity data classification

Of the 44 included papers, 13 (29.5%) included self-reported or prescribed ethnicity in medical records, 19 (43.2%) included self-reported ethnic groups with varying number of options (6 did not describe how many ethnic categories were provided, or if free-text was used to record ethnicity), 7 (15.9%) included interview-rated or prescribed ethnicity based on criteria such as appearance, family origin, names and language spoken at home (1 study did not specify what criteria is used), 2 (4.5%) used name-based computer algorithms to prescribe ethnicity, and 3 (6.8%) did not describe how ethnicity was asked (Table 3). No studies used free text to prescribe ethnicity.

**Table 3:** Source of ethnicity data classification by timeframe of publication

Source of classification	1946–2000	2001–2019	2020–2022	Total
Total Number of Papers	3	29	12	44
Self-reported (excluding medical records)				
4 choices	0	2 (6.9%)	0	2 (4.5%)
7 choices	0	0	1 (8.3%)	1 (2.3%)
8 choices	0	1 (3.4%)	0	1 (2.3%)
9 choices (1991 ONS)	0	3 (10.3%)	0	3 (6.8%)
16 choices (2001 ONS)	0	2 (6.9%)	1 (8.3%)	3 (6.8%)
18 choices	0	0	2 (16.7%)	2 (4.5%)
20 choices	0	1 (3.4%)	0	1 (2.3%)
Did not report	0	6 (20.7%)	0	6 (13.6%)
Interview Rated/Prescribed				
Based on appearance and grandparental origin	0	1 (3.4%)	0	1 (2.3%)
Based on family origin	0	1 (3.4%)	0	1 (2.3%)
Based on self-report, place of birth and parental place of birth	0	1 (3.4%)	0	1 (2.3%)
Based on language spoke at home	0	1 (3.4%)	0	1 (2.3%)
Based on names	1 (33.3%)	0	0	1 (2.3%)
Based on Mother's birthplace	1 (33.3%)	0	0	1 (2.3%)
Did not specify	0	1 (3.4%)	0	1 (2.3%)
Medical Records (self-reported or prescribed)	0	6 (20.7%)	7 (58.3%)	13 (29.5%)
Other				
Algorithm (based on forename-surname pairs)	0	1 (3.4%)	1 (8.3%)	2 (4.5%)
Did not describe how ethnicity was asked				
	1 (33.3%)	2 (6.9%)	0	3 (6.8%)

Missingness, Other, and Mixed (Table 4).

**Table 4:** Other, missing and mixed, by timeframe of publication

Other, missing, mixed	1946–2000	2001–2019	2020–2022
Total Number of Papers	3	29	12
Missing			
No missing	1 (33.3%)	10 (34.5%)	1 (8.3%)
Included missing	0	3 (10.3%)	2 (16.7%)
Reclassified	0	1 (3.4%)	1 (8.3%)
Treat as Other	0	1 (3.4%)	1 (8.3%)
Treat as White	0	3 (10.3%)	0
Excluded missing	0	4 (13.8%)	7 (58.3%)
Unknown	1 (33.3%)	2 (6.9%)	0
NA	1 (33.3%)	5 (17.2%)	0
Other			
No Other	0	0	0
Included Other, independent	1 (33.3%)	11 (37.9%)	9 (75%)
Included Other, clustered	0	5 (17.2%)	3 (25%)
Excluded Other	0	5 (17.2%)	0
Unknown	1 (33.3%)	4 (13.8%)	0
NA	1 (33.3%)	4 (13.8%)	0
Mixed			
No mixed, mixed is an option	0	0	0
Included Mixed, independent	0	3 (10.3%)	3 (25%)
Included Mixed, clustered	0	7 (24.1%)	5 (41.7%)
Excluded Mixed	0	6 (20.7%)	0
unknown	1 (33.3%)	8 (27.6%)	3 (25%)
NA	2 (66.7%)	5 (17.2%)	1 (8.3%)

### Missing data on ethnicity

Twelve papers (27.3%) reported no missing ethnicity, 12 (27.3%) included a missing group in their description or analysis (5 included as “missing”, 2 reclassified as other ethnic categories based on additional information, 2 grouped with “other”, 3 treated as “white”), 11 (25%) excluded missing group from analysis, 3 did not report sample demographics, and 6 (13.6%) were not applicable (ethnicity was prescribed, and missing is not considered as an option).

### Use of an Other ethnicity group

Twenty-one (47.7%) papers included “other” in description or analysis as an independent group, 8 (18.2%) included “other” but clustered it with other ethnic groups, 5 (11.4%) excluded “other” from analysis, 5 (11.4%) did not report methods of asking or reporting ethnicity, and 5 (11.4%) were not applicable (the assigned groups did not include an “other” option).

### Use of a Mixed ethnicity group

Six papers (13.6%) included “mixed” in description or analysis as an independent group, 12 (27.3%) included “mixed” but clustered it with other ethnic groups, 6 (13.6%) excluded “mixed” from analysis, 12 (27.3%) did not report how ethnicity was asked, and 8 (18.2%) were not applicable (the assigned groups did not include a “mixed” option). In all studies that provided a “mixed” option, this group was used by at least some participants.

### Justification for aggregating ethnicity

Of the 44 papers, 13 (29.5%) aggregated ethnicity when describing or analysing ethnicity and provided a justification (4 self-reported, 3 prescribed, 6 medical records), 14 (31.8%) aggregated did not provide a justification (6 self-reported, 3 prescribed, 5 medical records), 9 (20.5%) did not aggregate ethnicity (6 self-reported, 1 prescribed, 2 medical records), 2 (4.5%) used algorithm-based of deriving ethnicity, and the remaining 6 (13.6%) papers did not provide enough information on how ethnicity was asked or recorded.

Of the 13 papers mentioning a reason for aggregation, 3 (23.1%) reported minimising disclosure risks, 7 (53.8%) reported the need to avoid small subgroups or under numeration in analysis, and 3 (23.1%) provided a theoretical underpinning of why ethnicity was grouped.

### Analysing and theorising ethnicity

Of the 44 papers, 11 (25%) used ethnicity as a descriptor for the population, and 33 (75%) used ethnicity as a predictor for health outcomes. Of the 33 papers, only 8 (24.2%) explicitly theorised how ethnicity might explain differences in health outcomes.

### Focus group discussions

**Fig. 2:**
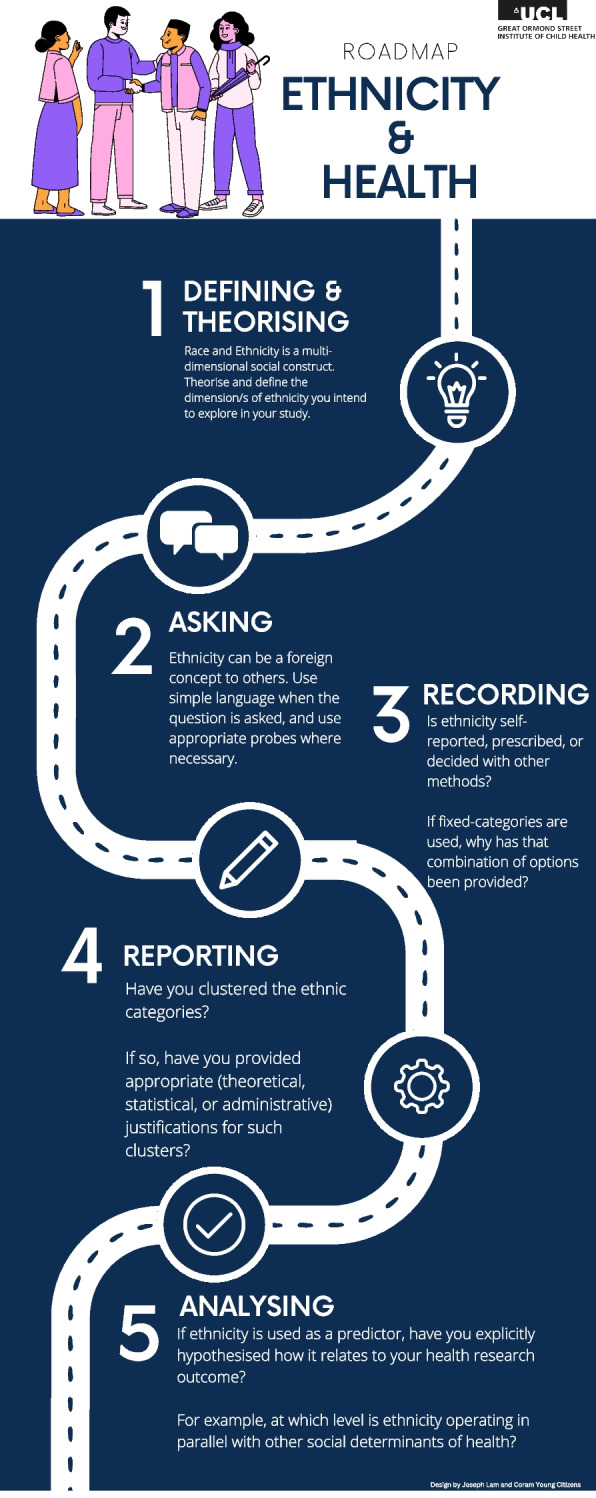
Roadmap for researchers to study ethnicity and health

A group of 10 young people from Nigeria, Turkistan, Syria, Yemen and Iran attended all 3 focus groups. Recommendations from our focus group discussions that could help researchers to record and analyse ethnicity information are summarised in Table 5.. Figure 2 provides a visualisation of the steps researchers should consider when designing surveys to capture ethnicity, or to analyse relationship between ethnicity and health.

**Table 5.**
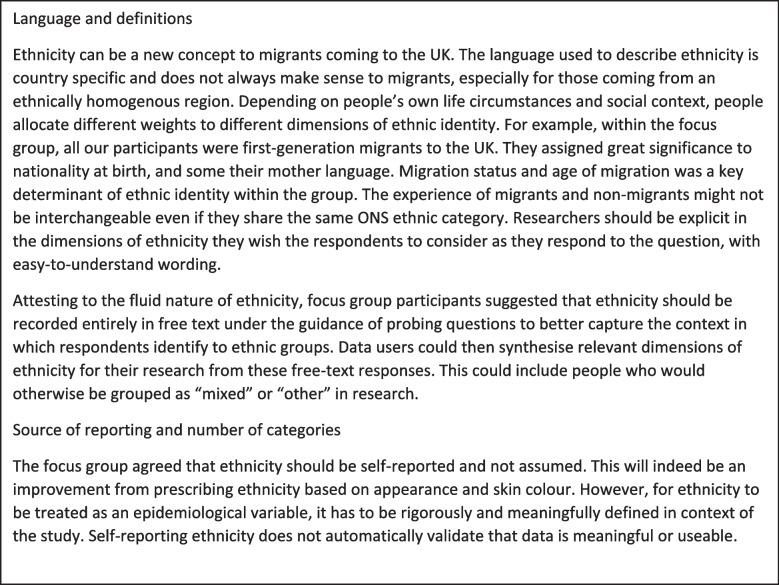
Recommendation from focus group discussions

## Discussion

In this bibliographical review, we found that in the most highly cited population health papers in the UK, researchers used inconsistent ways of asking and reporting ethnicity. Numbers of ethnic groups used across studies varied, and there was a lack of justification for aggregating ethnic groups into binary or high-level categories. Less than a quarter of papers modelling ethnicity explicitly theorised how it related to health outcomes. The focus group highlighted the need to adopt an accessible and clearly defined language to describe and capture the multidimensional and dynamic nature of ethnic identity.

This review consolidates the Race Equality Foundation’s [11] recommendation to close the ethnicity data gap. We extended the focus beyond how ethnicity is asked, and onto how ethnicity is theorised and analysed. Our review supports findings from a recent systematic review that found that ethnicity was inconsistently reported and represented in studies of multiple long-term conditions [38]. Our study highlights that the ethnicity data gap exists at both levels of inclusion and interpretation, and that our current data and evidence generation systems, from data curators to analysts, require more transparent and meaningful ways to represent ethnicity. For example, despite efforts to update methods of capturing ethnicity (moving away from merely separating “White and “coloured” populations [4]) our current operationalisation of ethnicity remains white-centric. This was evident in the lack of non-white mixed groups as an option in all of the studies we reviewed. The same pattern of white centrality persists in data analytical practices. All ethnic groups apart from “White” were subjected to aggregation and/or exclusion. Associations between ethnicity and health are understood in relation to the association between “White” groups and health outcomes.

We found a lack of explicitly stated theory in recording, aggregating, and analysing ethnicity. Lack of stated theory is not evidence of no theory, but a covert support of an essentialist model of ethnicity that rejects identity fluidity [40]. The essentialist model of ethnicity denies the lived experience that people can have multiple concurrent ethnic identities, over time or contexts. Focus group participants unanimously agreed that ethnicity changes over time according to personal experience, social pressure, and nationality changes. The essentialist framework denies the opportunities for data systems to capture and understand these changes. Instead of describing why a particular ethnic identity was reported, current strategies to harmonise or “clean” ethnicity recorded in administrative data further undermine the interpretability of ethnicity. Researchers analysing electronic health records or linked administrative health data lack autonomy in deciding how ethnicity is captured and recorded. However, researchers should develop insight in how ethnicity has been collected, to what extent researchers can draw inference, and clearly communicate the assumptions on what these ethnicity data may represent or misrepresent.

Census ethnic classification is used as the gold-standard measure across government and health records. In our review, several cohort studies used and referenced the same census ethnicity measures in their study to allow easier comparison to the UK population. However, it is not the language, but “how “ ethnicity is theorised that allows fair comparison. For example, being classed as “black” by health workers based on skin colour is not the same as having a subjective identity as “black” that stems from deep resonation with the black community’s struggles and group identity. Triangulating from the focus group discussion, an example of potentially invalid comparisons of ethnicity may be due to the non-interchangeable lived experience of migrants and non-migrants, even if they share the same census ethnic category. The problem is less about the sources of identification (whether they are self-reported or prescribed, or number of categories provided), but the need to clarify which dimensions of ethnicity are being asked about and captured, and whether they are being recorded faithfully in our data systems.

### Strength and Weakness

A major strength of this review was the involvement of marginalised voices from young people. They helped us ground our findings in light of their lived experience as minoritized ethnic groups and refugees navigating administrative and health systems in the UK. In this review, we focused on highly cited papers which are likely to have widespread impact on how other researchers report ethnicity and health outcomes. However, the review is limited by the non-systematic nature, and the relatively small number of papers synthesised. This restricts our ability to generalise the problems we describe. We relied on manual searches on Google Scholar, which is not a validated source of information, to record the number of citations for papers. Our focus group consisted of young people from migrant backgrounds and may represent different lived experiences compared to people from ethnically minoritized groups who have grown up in the UK. Further work with non-migrant populations would provide more insight, for example, in triangulating the generalisability of the recommendations from our focus group. Implementing what the focus group suggested in terms of capturing ethnicity (e.g., via free-text) remains challenging in administrative data systems. Balancing meaningful data collection, categorisation and analysis of ethnicity data with the cost of re-structuring, storing and safeguarding potentially more disclosive personal data will be a key consideration for administrative data controllers.

## Conclusion

The call for ethnicity to be treated as an epidemiological variable is not new. Senior and Bhopal [41] recommended that “each method of classifying ethnic groups should be recognised, and reports state explicitly how classifications were made”, and its “complex and fluid nature… widely appreciated”. We live in a rapidly diversifying world: continuing our normative approach in constant reviewing and renewing the number of ethnic categories is not sustainable nor meaningful, if these new categories are eventually clustered as “other” and fail to translate to health policies. Transparent, rigorous, and justifiable treatment of ethnic data is the cornerstone to improving data quality and developing insight to tackle ethnic health inequity, and an ethical responsibility to the people providing their data. After close to 30 years of venturing, now is the time for ethnicity to be freed from its shackles of being an administrative variable and embraced by researchers and the public as a meaningful epidemiological, research-viable variable. We can only start to tackle ethnic health inequity by treating ethnicity as rigorously as any other variables in our research.

### Supplementary Information

**Additional file 1. **

**Additional file 2. **

**Additional file 3. **

**Additional file 4. **

## Data Availability

The list of papers generated during the current review are available in JL’s Open Science Framework repository, https://osf.io/35rdc/?view_only=3e39f7099efb4e70b31f380faf432ea2, along with materials used to facilitate the public engagement focus groups. The papers analysed during the current study are included in this published article [and its supplementary information files].

## Abbreviations

UK: United Kingdom
ONS: Office for National Statistics
Covid-19: Coronavirus Disease 2019
PROSPERO: International prospective register of systematic reviews
PRISMA: Preferred Reporting Items for Systematic Reviews and Meta-Analyses
