# Supplementary material for: How is ethnicity reported, described, and analysed in health research in the UK? A bibliographical review and focus group discussions with young refugees
Source: BMC Public Health. 2023 Oct 17;23:2025. doi: 10.1186/s12889-023-16947-3 (PMC10583485; doi:10.1186/s12889-023-16947-3)
Supplement: Supplementary file 1 — Additional file 1. [file 12889_2023_16947_MOESM1_ESM.docx]

Additional File 1. Search Terms

The search strategy was developed by JL, then reviewed by a librarian with expertise in conducting bibliographical reviews.

Ovid MEDLINE(R) <1946 to July Week 5 2022>

| Search | Terms | Results |
| --- | --- | --- |
| 1 | exp Case-control studies/ | 1340523 |
| 2 | Epidemiologic Studies/ | 9145 |
| 3 | exp Cohort Studies/ | 2376251 |
| 4 | (epidemiologic adj (study or studies)).ab,ti. | 26287 |
| 5 | case control.ab,ti. | 128272 |
| 6 | (cohort adj (study or studies)).ab,ti. | 241959 |
| 7 | cross sectional.ab,ti. | 380044 |
| 8 | cohort analy$.ab,ti. | 9214 |
| 9 | (follow up adj (study or studies)).ab,ti. | 49805 |
| 10 | longitudinal.ab,ti. | 254604 |
| 11 | retrospective$.ab,ti. | 798446 |
| 12 | prospective$.ab,ti. | 724914 |
| 13 | (observ$ adj3 (study or studies)).ab,ti. | 211150 |
| 14 | adverse effect?.ab,ti. | 157617 |
| 15 | Cross Sectional Studies/ | 434508 |
| 16 | 1 or 2 or 3 or 4 or 5 or 6 or 7 or 8 or 9 or 10 or 11 or 12 or 13 or 14 or 15 | 3676764 |
| 17 | population groups/ or racial groups/ or ethnicity/ | 93678 |
| 18 | ethnicity.mp. | 127732 |
| 19 | "Ethnic and Racial Minorities"/ | 359 |
| 20 | "cohort profile".mp. | 832 |
| 21 | 17 or 18 or 19 or 20 | 150366 |
| 22 | exp Great Britain/ | 385633 |
| 23 | (national health service* or nhs*).ti,ab,in. | 210253 |
| 24 | (english not ((published or publication* or translat* or written or language* or speak* or literature or citation*) adj5 english)).ti,ab. | 38189 |
| 25 | or/22-24 | 574162 |
| 26 | (exp africa/ or exp americas/ or exp antarctic regions/ or exp arctic regions/ or exp asia/ or exp oceania/) not (exp great britain/ or europe/) | 3223405 |
| 27 | 25 not 26 | 560036 |
| 28 | 16 and 21 and 27 | 3200 |

Web of Science search terms

| Search | Terms | Results |
| --- | --- | --- |
| 1 | TS=case-control studies | 146852 |
| 2 | TS=epidemiologic stud* | 57889 |
| 3 | TS="cohort stud*" | 294926 |
| 4 | TS=cohort stud* | 694042 |
| 5 | TS=cross sectional | 528732 |
| 6 | TS=follow up | 1549108 |
| 7 | TS=longitudinal | 520549 |
| 8 | TS=retrospective | 669142 |
| 9 | TS=prospective | 726998 |
| 10 | TS=observ$ stud* | 134030 |
| 11 | TS="adverse effect" | 42800 |
| 12 | #1 OR #2 OR #3 OR #4 OR #5 OR #6 OR #7 OR #8 OR #9 OR #10 OR #11 | 4027258 |
| 13 | TS = ethnicity OR TS = "Ethnic and Racial Minorities" OR TS = "population groups" OR TS = "racial groups" OR TS = "cohort profile" | 145846 |
| 14 | TS = Great Britain OR TS = nhs OR TS = "national health service" OR TS = (english not ((published or publication* or translat* or written or language* or speak* or literature or citation*) adj5 english)) | 422396 |
| 15 | TS = africa OR TS = Americas OR TS = Antarctic OR TS = arctic OR TS = asia OR TS = oceania NOT TS = "great britain" NOT TS = europe | 740367 |
| 16 | #14 NOT #15 | 415344 |
| 17 | #12 AND #13 AND #16 | 1546 |
