# Supplementary material for: How is ethnicity reported, described, and analysed in health research in the UK? A bibliographical review and focus group discussions with young refugees
Source: BMC Public Health. 2023 Oct 17;23:2025. doi: 10.1186/s12889-023-16947-3 (PMC10583485; doi:10.1186/s12889-023-16947-3)
Supplement: Supplementary file 3 — Additional file 3. [file 12889_2023_16947_MOESM3_ESM.docx]

Additional File 3. Extended background, methods and materials used for Coram Focus Group Public Engagement Project.

The public engagement project is support by a Beacon Bursary 2023. The Beacon Bursary is an annual competitive public engagement funding by UCL Engagement. My project was one of 11 funded projects in 2022-2023, titled Ethnic Health Inequities and Data Justice – A Conversation with Young People. The public engagement project is designed to capture public opinion and was not designed as a research project.

Due to the public engagement nature of the project, no ethics approval is required. No audio recordings or photos are taken during the focus group discussions.

The PowerPoint slides and material used for the sessions were uploaded on my OSF page (<https://osf.io/35rdc/>). Below I summarise how the games and activities were designed to inspire discussions in the group.

Game 1: Evolving Identity: Imaginary Case Study of Johnny and Jacky (day1.pptx, slide 4)


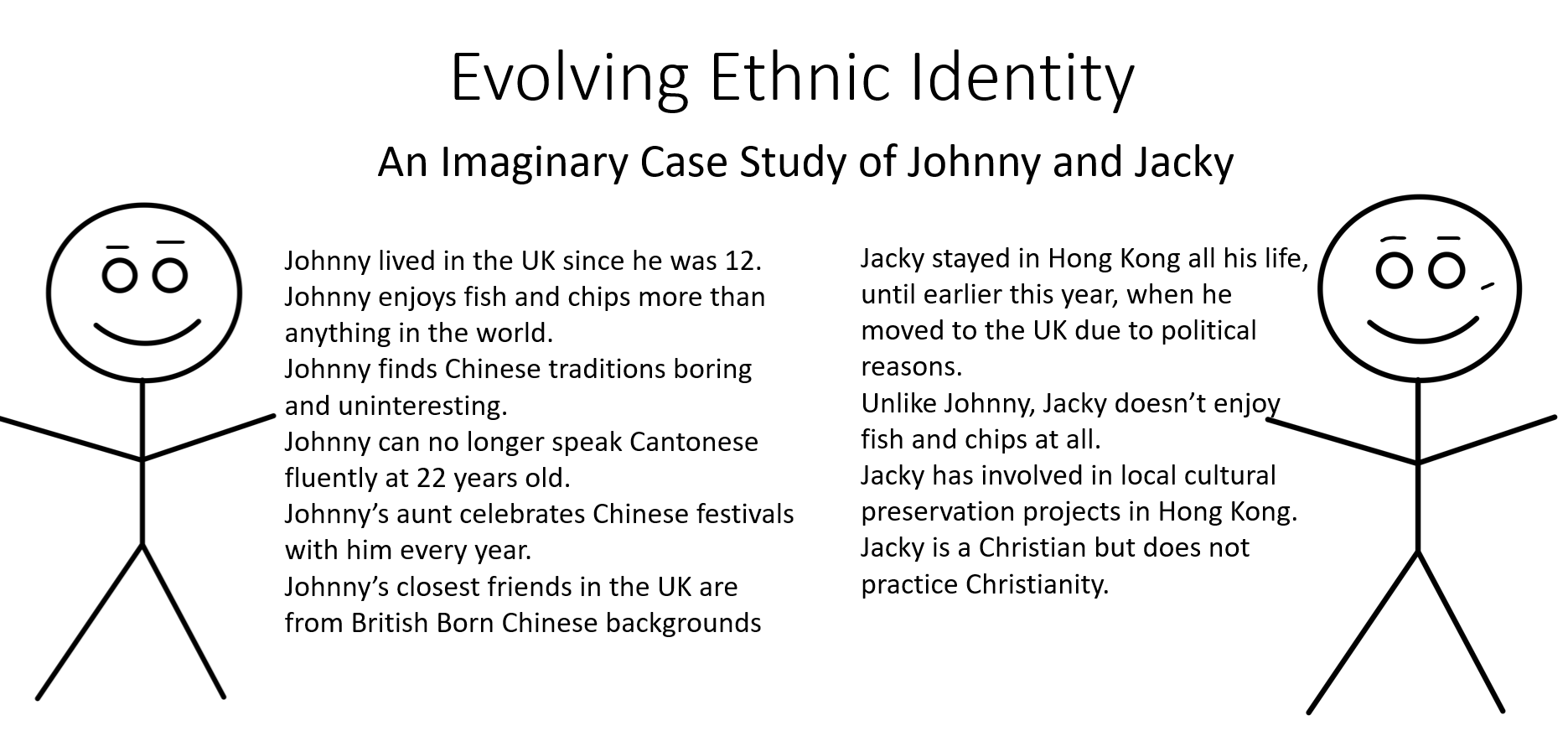


Additional figure 1. Slides used to illustrate the game (day1.pptx, slide 4).

This game is introduced as a thought experiment for the group to a recognise how a multitude of factors may contribute one’s ethnic identity, and the challenges for people to classify people into ethnic groups with limited information. The game is adapted in a poster presentation and captured conference attendee’s responses (additional figure 2). In both scenarios, there is an implicit assumption that Johnny (John) and Jacky are born to Asian parents. The big revelation that could be born of mixed heritage, or of White British heritage pointed our assumptions in considering ethnic identity.


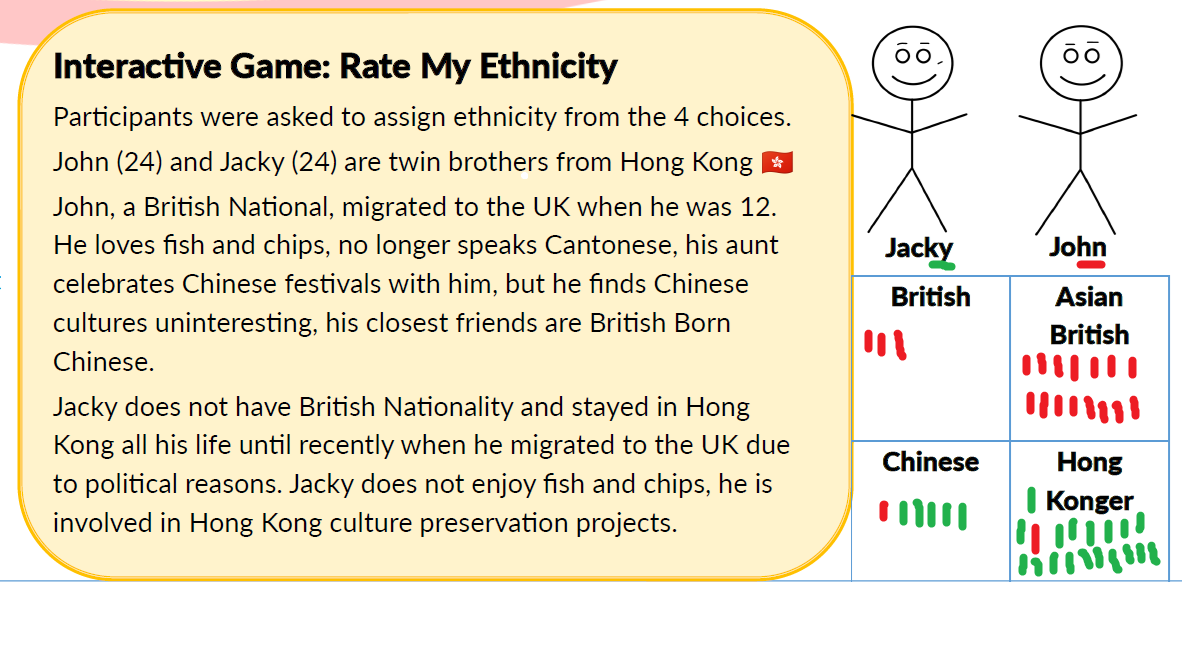
 Additional Figure 2. Adapted game presented at UCL Health of the Public Symposium 2023, with markings of conference attendee’s participation in the game.

Game 2: Tricky Categories (Day1.pptx, slide 5)

With reference to high level ethnic categories used by the Office of National Statistics (Asian, Black, Mixed, Other, White) in UK census, I asked the participants to prescribe ethnicities based on free-text descriptions I provided. The group is split into the Big-5 category group, and the 19-category group. Together we discussed whether there were consistency ratings across the 2 groups. This allowed participants to realise the benefits and costs of using such categories to classify ethnicity, in particular the lack of clarity in capturing other or mixed ethnic identities in the current paradigm.

Game 3: How should we ask about ethnicity? (Day2.pptx, slide 7)

In this game, I asked the group to design new ways for researchers to ask about ethnicity. The groups came up with alternative methods in which ethnicity can be captured and analysed. I described this in the main article box 1.

Other outputs from the focus group discussions

At the time of submission, members of the focus group have produced 3 blogs to capture their thoughts and reflections. The blogs are published on UCL Engagement Blog and the Administrative Data Research UK website. I have attached them at the end of this document. I hope these can offer a deeper insight into the group’s thoughts, told by their own words.

Blog 1: Ethnic Health Inequity and Data Justice – A Conversation with Abdulrahman (https://blogs.ucl.ac.uk/public-engagement/2023/05/10/ethnic-health-inequity-conversation/)

This article has been written by Abdulrahman Bdiwi, a young person from Coram Young Citizens . Young Citizens is Coram's award winning programme for 16-25 year olds from migrant and refugee backgrounds who make a difference to the lives of other young people new to the UK through direct work, improving practice and policy change. Find out more: https://www.coram.org.uk/what-we-do/our-work-and-impact/young-citizens/

Abdulrahman came from Syria and has lived in the UK for 5 years . This project has taken place with funding from The Wellcome Trust, and additional support from a UCL Beacon Bursary award.

Abdulrahman shared his reflections taking part in focus group discussions led by Joseph (Jo) Lam (Institute of Child Health), as part of a Beacon Bursary supported project: Ethnic Health Inequity and Data Justice – A Conversation with Young People. The project focus on exploring how ethnicity is understood, experienced, asked and recorded for young people from refugees and migrant backgrounds.

My opinion on the group discussion.

I have attended two sessions led by Jo from UCL at Coram to talk about ethnicity. Ethnicity wasn’t my cup of tea, I have never had any thought about it or even believed that it is something worth spending time looking at it. However, after just 15 minutes of the first session, I realised that ethnicity is something extremely important and equally complex.

The main challenge was that all my colleagues (who I worked closely with for over a year) have always found a way to agree on something at the end of our discussions, but not this time. It appeared to me that this topic has divided the team into different groups such as skin colour, political opinion or even religious beliefs. None of my colleagues (including me) were willing admit that our opinion might be wrong, or we might not have enough evidence to proof that we are right.

Things I have learnt from the discussion.

I have learnt that ethnicity is a complex issue, and there is no solution that would be fitting in all contexts. When we are discussing ethnicity, it is important to consider a range of factors, including ethnic group, place of birth, migration status, family history, and cultural background. These factors can all impact an individual's sense of identity and can help to provide a more understanding of the variety of experiences within any ethnic group.

Simultaneously, it is very critical to recognise that ethnicity is just one aspect of identity, and that individuals may identify with multiple ethnic or cultural groups. In addition, it is important to avoid making assumptions or stereotypes based on an individual's ethnicity, and to be aware of the possibility of discrimination or prejudice based on these factors.

Recording data about ethnicity groups is essential for supporting diversity and inclusion, understanding health disparities, and monitoring discrimination. However, there is a need to conduct this confidential information with care and sensitivity.

My opinion on the topic.

As a refugee myself, I totally believe that ethnicity is something very important as it reflects my identity and helps me to integrate into my new home without losing my family history and my cultural background. Also being aware of ethnicity adds significant value to the job market given the role it plays in encouraging diversity, which can lead to increased creativity, improvement, and productivity. However, I also believe that it could be used as a weapon against me and my existence in this county. If we are living in a country where refugees, migrants and other minorities are not protected by law, ethnicity is the easiest and most direct way for racist people to discriminate against other people, just because they have these data available for them. Therefore, ethnicity data must be used only for its aimed reasons, such as promoting diversity and inclusion, assessing health disparities, or monitoring discrimination.

When we talk about ethnicity, we should understand that the data collectors and the participants must have a clear understanding of the importance, advantage, and disadvantages of this sensitive data. When researchers record ethnicity data, it is critical to use a respectful and inclusive approach. We have to take into consideration, that not everyone will understand what ethnicity is. If you come from a country like mine where the vast majority are Arabs, such a question does not exist, so it was extremely hard for me to understand and answer such a question.

Ethnicity should be recorded in a way that respects the mixture of individual experience and acknowledges the complexity of identity. This might involve allowing individuals to self-identify their ethnicity, rather than relying on external categories or classifications. They should not make any assumption based in the appearance or name of individuals and allow them to choose the terms that best reflect their ethnicity and culture, even if they disagree with them. One way to achieve this is to offer a selection of options that reflect the diversity of ethnic identities and cultural backgrounds. For example, as a replacement of limited set of check boxes of categories, a more comprehensive approach could include an open-ended text field where individuals can enter the terms, they use to describe their ethnicity or cultural background. Researchers can derive relevant meaning from their descriptions.

My conclusion.

In closing, ethnicity is a vital aspect of a person's identity that can significantly influence their experiences and opportunities in society. Recording data about ethnicity groups is essential to encourage diversity and inclusion, understanding health disparities, and monitoring discrimination. On the other hand, it is essential to handle this sensitive information with care and sensitivity. By using a respectful and inclusive approach to recording ethnicity data, we can create fairer and more inclusive societies. Safeguards must be put in place to protect individuals' privacy and prevent misuse of this sensitive information.

Blog 2: Ethnic Health Inequity and Data Justice – A Conversation with Abdullahi (https://blogs.ucl.ac.uk/public-engagement/2023/05/18/ethnic-health-inequity-and-data-justice-a-conversation-with-abdullahi/)

This article has been written by Abdullahi Yussuf, a young person from Coram Young Citizens. Young Citizens is Coram's award winning programme for 16-25 year olds from migrant and refugee backgrounds who make a difference to the lives of other young people new to the UK through direct work, improving practice and policy change. Find out more: https://www.coram.org.uk/what-we-do/our-work-and-impact/young-citizens/

Abdullahi came from Nigeria and has lived in the UK for 10 years. Abdullahi Yussuf is a Social Anthropology graduate from School of Oriental and African Studies (SOAS) at University of London. He’s a prospective Master’s student that will be studying Human Rights from September 2023. He was a producer for the Royal Albert Hall's Young Producers Programme. Where he produced an event called Licence To DV8 at the hall. Abdullahi volunteers at Coram, We Belong and has three caseworker roles in justice, immigration and advocacy at Hackney Migrant Centre (HMC). He has experience of being on a student advisory panel for IntoUniversity and was a member of both Hackney Youth Parliament and Young Speakers of Hackney. Outside his busy lifestyle, Abdullahi enjoys learning Spanish and is a massive fan of wrestling! Abdullahi's favourite quote: "When you fight for your dreams, your dreams will fight for you”.

This project has taken place with funding from The Wellcome Trust, and additional support from UCL Beacon Bursary award.

Abdullahi shared his reflections taking part in focus group discussions led by Joseph (Jo) Lam (Institute of Child Health), as part of a Beacon Bursary supported project: Ethnic Health Inequity and Data Justice – A Conversation with Young People. The project focus on exploring how ethnicity is understood, experienced, asked and recorded for young people from refugees and migrant backgrounds.

The Significance of Recording Ethnicity Background Accurately

What is ethnicity? Ethnicity is when an individual or a group of people have a unique and shared culture, language, religion, or language. In essence, ethnicity is a set of social traits that determines how the human race differs and is similar in various spheres of life. "Academics have attempted to find terms to describe ethnicity - I think Donald L. Horowitz explained it best in 1985, when he pointed out that ethnicity is an umbrella ideology that “embraces groups differentiated by colour, language, and religion; it covers ‘tribes,’ ‘races,’ ‘nationalities,’ and castes”. I saw the concepts of ethnicity categories in action during the focus groups. Horowitz (1985: 53) explained it best when he pointed out that ethnicity is an umbrella ideology that “embraces groups differentiated by colour, language, and religion; it covers ‘tribes,’ ‘races,’ ‘nationalities,’ and castes”. I saw the concepts of ethnicity categories in action during the focus groups, thus Horowitz's summary of ethnicity is very accurate. A focus group led by a UCL PhD student asked young people for their opinions on this urgent issue as part of its investigation into how researchers could enhance its statistics on ethnic categories. As a young migrant and African man, participating in the focus group opened my eyes to how simple it is for me to answer questions about my ethnicity because "Black African" is always an option. The realisation that everyone has ethnic categories that they identify with was the thing I took away from the debates that I will remember the most, though. For instance, I spoke to Ahmed about his experience after the end of both sessions, he said: “Partaking in these sessions, I’ve realised that whatever ethnic category I decide to choose is up to me, as it’s based on my experiences with where I’ve grown to love since my childhood ”.

Ahmed's experience serves as a reminder of the need for the healthcare sector to improve the way that ethnic categories are entered into their systems. I have filled out forms and noticed that there aren't many options available; they are frequently listed in the following order: Asian or Asian British, Black, Black British, Caribbean or African, Mixed or Multiple ethnic groups, White and other ethnic groups. This short list of ethnic groups serves as a good illustration of why it is crucial for the UK healthcare sector to not only expand the list but also more accurately record people's racial and ethnic backgrounds. Finding out how heavily the UK healthcare sector depends on the "other ethnic group" option to appropriately match ethnic group data was one of the sessions' major takeaways for me. To accurately submit data and fight for justice, this component needs to be improved. Contrarily, this presented a problem during the sessions since the other participants and I realised how much effort has to be done to enhance data input for ethnic categories in the healthcare industry. The focus group, however, was a step in the right direction for me because I learned a lot more about ethnic groupings than I normally would. It was fascinating to learn more about the methods used on the ethnic groupings throughout the survey phase. As one's ethnicity is an important component of what makes one human, I believe the data on ethnicity should be documented more accurately and responsibly. In my perspective, switching people's ethnicities to the other option feels like eradicating a crucial piece of information on an individual.

I believe that listing all of the potential ethnic categories and leaving the "other" option open will enable the data on ethnicity to be collated in a way that is more inclusive and more in line with what the term "ethnicity" actually means. The fact that not everyone may be able to understand their ethnic backgrounds or that there may be language barriers to understanding it is another element that might be taken into consideration. By establishing the ethnic categories in multiple languages, the healthcare system can begin to significantly record ethnic backgrounds in a courteous, accurate, and acceptable manner. Given the amount of work the UK Home Office invests into ensuring interpreters are available for every stage of an immigration process, i.e. Migrant Help, I am confident that this investment will help to enhance the healthcare system in the UK. In the future, ethnicity records should, in my opinion, be treated with respect, given prime priority, and given options for multiple languages. People may start to have a newfound confidence in the healthcare system as a result, and a more inclusive modern society may also start to emerge.

Participating in a focus group that led this conversation was unquestionably a fantastic experience since it gave me a platform to openly share my opinions on this universal issue and it helped me better understand ethnicity and ethnic classifications. The fact that I had the opportunity to reflect and recognise how fortunate I am to be able to grasp ethnic groups while participating in the focus group and writing this blog made me raise the question: how those who have a language barrier would understand this topic? This is a key reason why I recommended language options when it comes to understanding ethnic groups as this will minimise the use of the ‘other’ option and will enable the healthcare system to record more accurately.

Having a youth voice in a global matter is extremely crucial as today’s youths are powerful, well-informed and determined change-makers. After all, youth and children account for over 40% of the global population, which is one of many reasons why the youth voice is so important. Having the youths’ voice during the focus group was not only the right thing to do, but it was also the tactical thing to do, as we are all striving for society to continue to be evolving rather than stagnate. It is now up to young people to shift the narrative of how things currently are to how they should be. Because this is a problem that affects young migrants, we are frequently the community that is seldom reached, thus I appreciated that the UCL PhD student worked with us during the focus group. Anyone organising a focus group with young people should be transparent and honest about the focus group's purpose, how the participants' ideas will contribute to the overall objective, and how the participants' privacy will be maintained. Once this is implemented throughout the entire process, the individual will understand that the voices and opinions of young people possess the strength, agility and a wide range of talents to bring about the constructive changes we want to see. I advise anyone organising a focus group to invest in amplifying and hearing the views of young people because it helps us feel like we belong, builds our self-esteem, and develops our leadership skills, all of which are necessary for both our professional and personal life.

Blog 3: Public perspectives on ethnicity in administrative data research (https://www.adruk.org/news-publications/news-blogs/)

Administrative data is about the public. When it is used for research, there is a duty engage the public in how their data is being used. In this blog we hear directly from three members of the public about their perspectives on the use of ethnicity in research using health and administrative data.

Members of the Coram Young Citizens, Lavin, Benhnam and Rakiba, discuss and reflect on their experience taking part in a series of focus group discussions with young people from a refugee and asylum-seeking background. This is part of research led by UCL PhD student Joseph Lam (Jo), which aimed to understand their perspectives on the use of ethnicity in research using health and administrative data.

The focus groups included 10 young people originally from Nigeria, Sudan, Syria, Iran, Kurdistan and Iraq. They attended two sessions in which they discussed the following questions:

1. What is ethnicity? How should ethnicity be asked inclusively and be better recorded?

2. Does ethnicity change over time and context? If so, why?

The problem of categorising ethnicity, as told by Behnam and Rakiba

Behnam: Mixed groups are often faced with difficulties in recording their ethnicity due to societal pressure to identify with a single race or ethnicity. They are given limited options that they either have to identify by their skin colour or the continent they’re from, and this mainly affects people of colour. For example, an Arab person can be identified as Arab, Asian or other.

Rakiba: One solution we discussed is leaving the ethnicity option open and letting people identify with the description that suits them best to ensure inclusivity and diversity. It is important to allow participants to self-identify more flexibly, respecting and celebrating diversity and recognising that there is no one-size-fits-all approach to ethnic identity.

Behnam: I’m Iranian and in Iran we don’t have any different ethnicities. We don't use this word at all - we see each other as Iranian, but at the same time, we have different ethnic languages and accents. The language used in the UK to describe ethnicity did not immediately make sense to me.

Problems arise when we must tick a box. Some of the categories don't make sense to me, like “Irish traveller” or “other Arab”, and they seem to forget to include some ethnicities there. I would identify as Asian: Iranian but this isn't an option and usually for most of the things I just chose other.

Rakiba: Ethnicity is a personal identity, and it is up to everyone to decide how they identify and what ethnicity means to them. In the beginning when I first came to the UK, I used to identify myself as two ethnicities - Irish and Syrian – in legal documents. However, the process of gathering evidence and legal documents to prove my identity was challenging. As my Syrian documents have expired, I’d rather identify as Irish only for a peace of mind and to avoid the fees, extra questions and extra evidence I would need to provide to identify as Syrian.

Changes in ethnicity over time, as told by Lavin

Living in a different region can expose a person to a different language, lifestyle, and culture, ultimately impacting their sense of identity. For example, if someone from an African country moves to the United States and learns to speak English fluently and adapts to American culture, they may no longer identify solely as African but rather as African American.

The focus groups suggested ways of responding to someone’s ethnicity changing in the data, Many suggested ways of treating changes in ethnicity assumed ethnicity does not change over time, such as choosing whichever ethnicity is reported more often or choosing whichever ethnicity is closest to the present. These methods assume that ethnicity does not change over time. At present, when someone’s ethnicity record is different across data sources, or over time, most researchers dealt with it by choosing the most recent, or the most commonly reported ethnic categories. These methods assume that ethnicity do not change over time. However, it is important to acknowledge that these methods may not work for everyone, and that individual experiences and identities may not fit neatly into existing categories. To address this, researchers should be asking more specific questions to explore potential reasons for a changing ethnicity, and how it could impact health.

This is a topic I had never considered before, but after listening to the other participants' ideas and thoughts, we have concluded that ethnicity can change over time.

Drawing conclusion

Migrants and non-migrants’ lived experience of ethnicity is not fully inter-changeable, even if they share the same Census ethnic category. Researchers should communicate clearly how ethnicity is operationalised for their studies using linked administrative data, with appropriate justification for clustering and analysis that is meaningfully theorised.

This work was supported by funding from the Wellcome Trust (212953/Z/18/Z) and a UCL Engagement Beacon Bursary. Young Citizens is Coram's award-winning programme for 16–25-year-olds from migrant and refugee backgrounds who make a difference to the lives of other young people new to the UK through direct work, improving practice and policy change. This is a young-persons-led blog.
